# The Southern Medical College

**Published:** 1879-10-20

**Authors:** 


### EDITORIAL AND MISCELLANEOUS.

### *THE SOUTHERN MEDICAL COLLEGE.*

This new Institution had a very flattering opening, on the 13th inst.

The number of matriculants was decidedly encouraging, being larger perhaps than that of any Southern School at its opening.

As the students had not all arrived it was decided to defer the regular didactic course until Monday, the 20th, the time to be profitably filled in the meanwhile by daily clinics and clinical lectures, between the hours of 9 and 12 daily.

A public introductory was delivered on Tuesday, the 14th, by Prof. Powell, on *Southern Institutions*, which was able, eloquent and well received. This institution is receiving material for an elegant museum, a library, and an excellent outfit of apparatus for all departments. We go to press before the first regular lecture in the institute, which will be delivered by the Professor of Physiology on Tuesday, 20th instant, 9 o'clock a.m. J.

